# COVID-19 in missiological and historical perspective

**DOI:** 10.1177/0091829620972386

**Published:** 2021-01

**Authors:** Benjamin L Hartley, Robert A Danielson, James R Krabill

**Affiliations:** School of Theology, Seattle Pacific University, USA; ESJ School of Worl Mission and Evangelism, Asbury Theological Seminary, USA; History, Theology and Ethics Department, Anabaptist Mennonite Biblical Seminary, USA

**Keywords:** Nigeria, pandemic, Floating Christian Endeavor, John R Mott, World War I, influenza, Antoinette Palmer Jones, African initiated churches, healing prayer, African Christianity

## Abstract

COVID-19 is affecting Christian mission in many different ways. Doubtless it is inspiring some people to initiate new mission efforts, while in other contexts it is causing thriving mission to change radically or cease altogether. In this forum article, three missiologists write essays about how mission was affected during the influenza pandemic of 1918–1919, the event most frequently compared to COVID-19 for its similarly worldwide scope. James Krabill’s essay describes how the earlier influenza pandemic led to renewed spiritual vigor in Nigeria and the establishment of several new denominations in West Africa, which remain influential today. Robert Danielson’s essay examines how a ministry to sailors in the early 20th century, known as the Floating Christian Endeavor, was negatively impacted by the influenza pandemic. This article concludes with Benjamin Hartley’s story of how the life of John R Mott, perhaps the most famous world Christian statesman in 1918, was also affected by the influenza’s scourge. These historical essays provide both inspiration and consolation for contemporary mission initiatives as missiologists and other Christian leaders seek to respond to the crises of their own day.

When the American Society of Missiology decided to cancel its face-to-face 2020 annual meeting in Indiana some months ago, one of the first questions raised was: “How, then, should we respond?” COVID-19 has, after all, changed how Christians are doing ministry in profound ways, and the full effects of how mission is changing remain to be seen. Practitioners and scholars alike find themselves asking hard questions about this new context to which they are called to be in mission.

In the early weeks of the pandemic’s spread, one heard the term “unprecedented” used a great deal in the press and by national and international spokespersons. Pandemics like the one we are experiencing, however, are not in the least bit unprecedented, but rather quite common in world history. Epidemics and pandemics have also shaped the way mission happens for a very long time.

We may not know precisely how we should best respond to our current pandemic, but the testimony of forbears in mission is surely a place to turn for both comfort and inspiration. In the early church, Christians’ self-sacrificial acts in caring for the sick during the 6th century’s bubonic plague outbreak were a way they made clear just how central love was for this new, strange movement of Jesus followers (Eusebius, in Reff, 2004: 74). During the 1793 yellow fever epidemic in Philadelphia, African Americans were (wrongly) believed to be immune from the fever. The future founder of the African Methodist Episcopal Church, Richard Allen, mobilized his followers to care for the sick, regardless of race. In the process, Allen won over many whites in his work for racial justice in that city (Newman, 2008: 87–89). When the first missionaries reached the northwest coast of the USA in the early 1830s, they found themselves in the midst of a malarial epidemic (brought by ships sailing north from South America), which killed up to 85% of the Native American peoples in the region (Boyd, 1996: 141). The missionaries’ first ministry was to establish a makeshift orphanage for the children of the epidemic’s victims.

The 1918–1919 influenza pandemic is the historical event with which most people are drawing comparisons for the challenge we face today from COVID-19. In this forum article, we examine three different examples in mission history of missionaries—both western and indigenous—who grappled with the effects of the so-called “Spanish flu.” James Krabill’s essay illustrates how the pandemic prompted new missional fervor and led to the establishment of several indigenous church movements in West Africa. Robert Danielson tells the story of how the pandemic more negatively impacted the ministry of the Floating Christian Endeavor Society, a ministry to sailors. Benjamin Hartley’s essay on John R Mott highlights several different ways that the 1918–1919 pandemic influenced Mott’s personal life, diplomatic efforts, and fundraising goals. For all of these cases (and many more not discussed here), the pandemic’s influence raises intriguing counterfactual and missiological questions. What would have happened if the pandemic had *not* been a factor or had been responded to differently? Would some new directions in ministry not have taken place? How did the realization of human vulnerabilities in the face of the impacts of war and the flu affect how Christians understood mission? As we consider our own context, what are the key questions that missiologists need to be asking themselves now? It is our hope that these stories of Christian mission in the midst of a pandemic will inspire us all to reflect deeply on our own context as we seek to be faithful participants in the mission to which we have been called.

## The 1918–1919 influenza pandemic: the flame that ignited religious innovation in Nigeria

### James R Krabill

In an August 2020 syndicated column, the *Philadelphia Inquirer* journalist Alfred Lubrano asked whether COVID-19 will “inspire the faithful” and create a spiritual surge. Lubrano noted the horrific impact of the 14th-century Black Death on Europe. The death of 25–30 million people drained the faithful of faith in God, who, many believed, had “failed to hear the prayers of the doomed” (Lubrano, 2020: B3).

Lubrano believes that the current pandemic will be different. He cites Greg Sterling, Dean of Yale Divinity School, who states that “this pandemic is making everyone acutely aware of the fragility of life. . . . People are spiritual, and I think the need to connect to God may be greater after this is over” (Lubrano, 2020: B3). A surge of interest in spiritual things is precisely what happened in Lagos, Nigeria, during the 1918–1919 influenza pandemic just over a century ago.

With a total number of estimated deaths worldwide at 50 million, the influenza pandemic hit Lagos on 14 September 1918 and spread with devastating speed. Lagos lost 1.5% of its 81,941 inhabitants. Beginning in the ports among seamen, the virus quickly infected most people in Lagos Island and moved from there to the interior. The British colonial administration attempted to educate people on how to remain healthy and put in place a house-to-house disinfection program across the city of Lagos. But city-dwellers fled Lagos—often by train, the major means of transportation at the time—and carried the virus with them to other parts of the country. Within three months, the highly contagious disease had spread across Nigeria, and it continued unabated until mid 1919, when those who had been infected either died or developed immunity to the virus (Adebowale, 2020).

At the peak of the crisis, prayer bands formed in southwestern Nigeria and eventually developed into a religious movement under the general name of *aladura*, a Yoruba term for “the prayer-people.” The focus of these groups was fasting, visions, dream interpretation, prayer protection, moral conduct, and healing without medication—traditional or western. By 1950, the *aladura* churches emerging from this initiative were the most prominent Christian expression in the country, with considerable influence in Nigerian society (Kim and Kim, 2008: 82).

There were, of course, multiple impulses that gave rise during this time of colonial rule to the advent of more indigenous forms of Nigerian Christianity. The wave of “Ethiopianism” that spread across Africa from 1860 to the end of the 19th century was clearly a response to western imperial Christianity. Jehu Hanciles (2002) traces this trend toward indigeneity and independence to Anglican mission theorist and administrator Henry Venn, and the confidence Venn placed in Africans’ ability and capacity to run their own churches. Ogbu Kalu (2007: 269) emphasizes Venn’s important role in counseling expatriate missionaries to “build nuclei congregations, study and respect indigenous peculiarities, and avoid mistaking black nationalism for presumption or ingratitude.”

Whatever progress was made by Venn and others in the mid 19th century was dramatically altered after the Berlin Conference of 1885 and the resulting partition of Africa among European powers. Vicious forms of European national fervor descended on the continent. Respect for local ruling chiefs and belief in African capacity disintegrated. The attitudes of missionaries in the growing wave of arrivals from Europe were increasingly shaped by an Enlightenment world view, with disparaging views of Africa. Some of the newer, younger missionaries openly disdained educated “black Englishmen” and championed paternalistic policies that favored hierarchy, discipline, and control (Kalu, 2007: 261). Not surprisingly, Africans responded with increasing rejection of European insults, racism, missionary polity, cultural domination, and religious control. A Nigerian Southern Baptist leader, David Brown Vincent, began “wearing only Yoruba clothes, founded a school with no foreign support, and in 1888 seceded from the Southern Baptists to form the Native Baptist Church in Lagos, the first indigenous church in West Africa” (Kalu, 2007: 271). Other leaders and revival movements followed suit, splitting from mission-founded churches, rejecting their European baptismal names, offering prayers for local chiefs rather than British royalty, and accepting into church membership men and women in polygamous marriage relationships.

The catastrophic impact of World War I further distanced Africans from their European rulers. Condescending attitudes and racist policies—including those employed and expressed by the missionary community—had already alienated many in the local population. Then came the “Great War,” with Africans witnessing colonial powers shamelessly and aggressively seeking to invade and occupy territories of their imperial competitors. As Robert O Collins writes:

The war’s insatiable appetite for raw materials to feed Europeans and their factories necessitated unprecedented demands on Europe’s African subjects. . . . All of the colonial governments arbitrarily requisitioned men into service as soldiers and laborers. . . . Those soldiers and workers who survived the fierce fighting in Europe often returned to African village life no longer in awe or fear of their colonial rulers. Many of them had witnessed the unprecedented scenes of Europeans killing Europeans in France and in Africa that inevitably undermined the moral authority, aura of invincibility, and the belief in the racial superiority of white Europeans. (Collins, 2006: 197–198).

Two months after the influenza pandemic struck Nigeria with full force, World War I ground to a halt with the signing of the 11 November 1918 armistice. The city of Lagos was “in a ripe state for religious innovations,” (Peel, 1968: 60) though the influenza attack had dealt it a mighty blow. One correspondent reported in the *Lagos Standard* on 23 October 1918: “Lagos has passed through terrible times the last two or three weeks . . . it is like a veritable city of the dead” (Peel, 1968: 60). The colonial government decreed that churches should close, crippling the religious activities of the community and paralyzing its spiritual life. Large numbers of job-seekers “raised a socioeconomic problem which they naturally hoped Providence, above everything else, could solve” (Omoyajowo, 1982: 4).

Apocalyptic prophecies abounded throughout the city. One self-proclaimed “Messenger to Ethiopians,” a certain Adeniran Oke, stirred up a panic by declaring that Lagos was about to be inundated by a tidal wave: “He went about the city in sackcloth and ashes, with bell and Bible, to preach the need for a religious revolution” (Peel, 1968: 61). Everywhere, people formed prayer groups, embracing prayer as the only alternative to western medicine’s apparent failure and impotence in the face of influenza. The earliest groups—considered unacceptable by church authorities—grew out of Anglican prayer meetings led by Joseph Shadare and Sophia Odunlami (Kim and Kim, 2008: 82). Shadare was seeking support in 1918 against the spreading influenza virus and formed a prayer group “as the result of a dream in which he saw the church divided into two groups, those who neglected prayer and those who resorted to prayer constantly” (Turner, 1967: 9). At the outset, this was an initiative taking place within the church with the minister’s supposed approval and support. Historically, according to Turner (1967: 9), this could be regarded as “the first of the *aladura* societies,” though several years would pass until any of these early “societies” would become independent of mission-founded churches and formally constituted as government-recognized entities.

A second development came about through one of Shadare’s relatives, Sophia Odunlami, a young schoolteacher at the time. Odunlami began to have—by her own testimony—inspirational Holy Spirit experiences, though some in the Anglican community interpreted such strange phenomena as devil possession and her father interpreted them as madness:

She herself had been stricken only lightly by the influenza, but she began to proclaim that anyone who contracted the plague would die if they trusted in the power of medicines, and would be healed if they relied only upon water from the rain that she prophesied would fall. (Turner, 1967: 9)

Odunlami eventually took up residence with Shadare, a goldsmith by profession, and shared in the emerging prayer-society activities known variously by a name revealed to Shadare in a dream as the “Precious Stone” or the “Diamond Society” (Turner, 1967: 9–10).

Over the course of time, three independent movements took shape in Nigeria from the scattered sparks of charismatic stirrings and devotion to prayer with origins during the 1918–1919 influenza pandemic—the Church of the Lord (Aladura), the Christ Apostolic Church, and various branches of the Cherubim and Seraphim Church. All three churches—which today have millions of active members—remain influential in Nigeria, across West Africa, and in major European and North American cities. In 1975, the Church of the Lord (Aladura) was one of the first African-initiated churches to become a member of the World Council of Churches (2020).

While the pandemic was the flame that ignited the smoldering embers resulting from physical trauma and mounting discontent with colonial rule, the emergence of these new movements also reflected broader trends spreading across the continent—trends that Nigerian church historian Ogbu Kalu has identified as

the quests to appropriate the gospel and modernity with dignity; to be both an African and a Christian; to express faith from an indigenous worldview and spirituality so that Africans could respond to their own realities and culture in the spheres of liturgy, polity, and ethics; to tap the resources of indigenous knowledge in communicating the kerygma; and to practice local initiatives in evangelism, decision-making processes, ecclesial structures and funding. (Kalu, 2007: 277)

With all of these factors at play, the stage was set at the beginning of the 20th century for change to happen. In Lagos and other Nigerian urban settings, however, European cultural patterns and expectations remained strong, and African beliefs were generally viewed as primitive and unprogressive. Then came the influenza calamity of 1918–1919, forcing people to seek local resources, both physical and spiritual, in confronting a crisis that outside solutions failed to address adequately. And, for that, growing numbers of Christians turned to prayer, prophetic counsel, angelic protection, dreams, visions, miracles, and healing—in short, to indigenous resources rarely referenced in mission-founded churches yet present in scripture and familiar to African sensibilities. Over the next few decades, the seeds planted during the pandemic gave way to religious innovation *by* Africans, *for* Africans, and in response to the needs *of* Africans, which continues to shape the face of Christianity in Africa today.

## Floating Christian Endeavor: how influenza helped kill a vibrant mission

### Robert A Danielson

Before there was the Student Volunteer Movement there was the Young People’s Society of Christian Endeavor. Commonly known as the Christian Endeavor Society, it was founded in 1881 at a Congregational church in Portland, Maine, as the first Christian youth organization. It sought to empower young people for service to the church, and soon bands of young laypeople were inspired to pursue ministry “for God and the Church” across the USA, Canada, and around the world (see Clark, 1895).

One early local society was founded at the First Congregational Church in Falmouth, Massachusetts, on the coast of Cape Cod. A young man associated with the church was Madison Edwards (13 August 1852–15 August 1926), who had been inspired to begin preaching to sailors on vessels in Woods Hole (the current home of the well-known Woods Hole Oceanographic Institution) when he was just 16 years old (Wiseman, *c.*^1978^). When he found that he needed additional assistance, he recruited the young people of the local Christian Endeavor Society to help. Antoinette Palmer Jones (20 November 1856–15 December 1918) was the secretary of the local society and also lived close to the telegraph office in Falmouth. When a need arose, Edwards would contact her by telegraph and she could mobilize the society to meet whatever problem had arisen (Showalter, 2011: 26).

In 1890, Edwards and Jones came up with an idea to establish Christian Endeavor societies onboard government ships. This way, the young sailors they brought to Christ could have a group for support, discipleship, and encouragement when they were away from the established ministries in the ports. Jones sent the idea to the Christian Endeavor headquarters in Boston, and the Floating Christian Endeavor was born (see Danielson, 2014). Showalter (2011: 26) points to church records where Antoinette Palmer Jones recorded the establishment of a society on 12 May 1890 on the Revenue Cutter *Dexter*. About a month later, Antoinette Palmer Jones was named superintendent of the Floating Christian Endeavor.

The organization was simple and relied on two distinct groups of laypeople. There were land-based societies in various ports, especially along the East Coast of the USA, and there were floating societies of sailors that formed on the ships themselves, which were transient (see Danielson, 2016). Jones (1895b) described the purpose of the Floating Christian Endeavor society when she wrote: “It emphasizes the brotherhood on the sea, the fellowship on shore, and makes a sailor responsible for a Christian man’s work wherever he may be, on man-of-war, steamship, merchantman, coaster or fishing vessel.” Members would organize their own meetings and committees, when possible, and hold each other accountable for their faithfulness. Local land-based societies were responsible for the shipboard societies they organized. Their job was to support the ministry to the sailors, hold meetings on land, and collect funds and items to support the work at sea (see *San Francisco Call*, 1897). These organized local societies corresponded with Antoinette Palmer Jones.

In the days before the Internet, communication with men on ships was difficult, yet the Floating Christian Endeavor society recognized the need to maintain contact with all of their members as much as possible. While Jones would begin her work with the Floating Christian Endeavor as the superintendent of the Floating Societies of the Christian Endeavor, by 1905 she was corresponding secretary of the Floating Societies of the Christian Endeavor. In 1908, at the formation of the World’s Floating Christian Endeavor Union, Jones was named president (Fegert, 1914). In essence, while this vast work relied heavily on societies to function at the local level, the main organization and its principle communication network both centered on the single person of Antoinette Palmer Jones.

Reforming the moral character of sailors at sea was a crucial goal of the floating societies. In a witty remark that was picked up and used by others, George Coleman (1901: 114) told the 1901 convention of the International Christian Endeavor that the Floating Christian Endeavor was like Ivory soap, which, in the words of the popular advertising phrase of the day, was “99 and 44/100 percent pure, and IT FLOATS!” Temperance, moral purity, and ending profanity were major religious goals of the organization. Jones is quoted in an article as saying that a sailor told her “five years ago it was a rare thing to find a Christian man in the naval service, but that now it was not a rare thing to see a Christian Endeavor pin on a sailor’s uniform” (*San Francisco Call*, 1897).

At the 21st convention of the International Christian Endeavor, Navy Chaplain Robert E Steele pointed out that Floating Christian Endeavor was not about mission *to* sailors, which had been tried in the past, but about the mission *of* sailors to other men of the sea, which once again highlights the lay-led nature of this “church” upon the sea (United Society of Christian Endeavor, 1903). The entire concept was to empower laymen who were sailors to evangelize other sailors at sea, and thus enlarge the Church in the process. Francis Clark (1906: 468), the founder of Christian Endeavor, also pointed out that, at the time, this was the only major religious work among sailors in the navy.

During the Spanish–American War (April–August 1898), the Floating Christian Endeavor became even more prominent. The society on the USS *Charleston* had helped found the Christian Endeavor Seaman’s Home in Nagasaki, Japan, and also provided one of the early martyrs of the group, who had become part of the Floating Christian Endeavor society on the USS *Maine* before it was sunk in Havana, Cuba, on 15 February 1898 (Coleman, 1901: 115). There was a Floating Christian Endeavor society on the USS *Olympia* with Admiral Dewey when he fought the Battle of Manila Bay on 1 May 1898 (Coleman, 1901: 115; *The New York Times*, 1898).

There was a Floating Christian Endeavor society on the USS *Oregon* when, in 1898, she had to make a rapid journey from the Pacific Ocean to Cuba around the tip of South America (Coleman, 1901: 115). She did the journey of 13,675 nautical miles in 66 days, which was amazing for the day, and the feat was recognized nationally. The vessel then fought in the Battle of Santiago de Cuba and earned the nickname “The Bulldog of the Navy.” Following the Spanish–American War, there were at least two Floating Christian Endeavor societies on the *Vermont* and the *Nebraska* as part of President Theodore Roosevelt’s “Great White Fleet,” which traveled around the world from 16 December 1907 to 22 February 1909 (Cowan, 1908: 10).

In just a short span of time, the work had grown tremendously not due to the work of traditional missionaries or an ordained chaplaincy, but due to the flexibility of a lay-led movement, accompanied by the transient nature of the mission field and a genuine passion for Christ in its members. The movement quickly spread internationally as well, with Clark mentioning Floating Christian Endeavor societies on British and Japanese vessels. Antoinette Palmer Jones (1895a: 65) mentions societies on ships from Canada, New Zealand, and Australia, and one society “among the marines of the Imperial Japanese Navy, whose members in the recent war, went to the front, two of whom were killed.” In a report on the work of the Christian Endeavor Society among sailors and soldiers, one writer notes:

Think of the sailor’s reputation for wild living, remember the loneliness of his calling, his freedom from restraint when going ashore, and then let me tell you that over 150 floating societies of Christian Endeavor have been organized and more than 6000 sailor lads have taken their Christian Endeavor pledge. Now remember, too, a sailor’s opportunity for meeting many peoples, and imagine, if you can, what a force for righteousness 6000 sailor-men can exert in the world. (Coleman, 1901: 114)

However, the flexibility of the organization, which allowed it to adapt to life on the sea, was only loosely organized and connected to the oversight of Antoinette Palmer Jones. Without her at the helm, there was just a coalition of smaller local societies each doing their own work in a narrow geographical area. She does not appear to have had much of a staff or organization to support her work (or even a salary), even though she was held in high regard. In one report, recording an illustrated lecture being given, the writer notes:

The audience was particularly pleased with a fine picture showing, in her own home, which has been an altar of devotion to the welfare of the boys in our navy and merchant marine, Miss Antoinette Jones, Falmouth, Mass., the “sister” of every sailor afloat. (United Society of Christian Endeavor, 1903)

As the Floating Christian Endeavor movement was growing and thriving, it was hit with two simultaneous crises. First was the entry of the USA into World War I in 1917. As a movement working with sailors onboard US government ships, suddenly communication and organization became more complicated in wartime. However, it had earlier thrived during the Spanish–American War, so such challenges were not insurmountable. But a subtler enemy was about to emerge. In April 1918, cases of influenza appeared in a mild first wave in Haskell, Kansas, among soldiers in training for the war (see Barry, 2018). As soldiers traveled to Europe, the influenza traveled with them and mutated into a more virulent strain. On 28 August 1918, the first cases of a second wave of influenza broke out among sailors on a receiving ship at Boston’s Commonwealth Pier and, by September, had spread to the US Army’s training camp at Camp Devens outside of Boston (see University of Michigan Center, 2016). By mid September, around 2000 sailors were infected in Boston and the first civilian cases were occurring. Churches, theaters, public meetings, and events were closed down across the city. By the time the shutdown was lifted on 19 October 1918, around 3500 Bostonians were dead. But influenza continued to spread through the nation and through the state of Massachusetts.

On 26 September 1918, the town of Falmouth (just 100 miles south of Boston) closed all churches, libraries, schools, and places of amusement. In October, cases of influenza began to appear in Falmouth, spiking to 423 cases in December. This small town of about 3500 people reported a total of 595 deaths in 1918, as opposed to 65 in 1917 (see Nickerson, 2018; White et al., 2020). In the midst of this turmoil, Showalter (2011: 30) reports that, on 15 December 1918, Antoinette Palmer Jones died of influenza and her funeral was held at her home in Falmouth, since the churches were closed due to the epidemic. She was just 62 years old and, while her official death record reports her death in Boston of “myocardial degeneration,” this underlying health condition was most likely exacerbated by influenza. The *Christian Endeavor World* (1919), the official paper of the Christian Endeavor Society, noted in her obituary: “She had been failing in health for several months, but up until the week of her death had continued her work for the sailors and soldiers.” Whether Jones caught influenza from a sailor or from her work with the Christian Endeavor Society headquartered in Boston will never be known, but the impact of her death on the Floating Christian Endeavor was terminal. The organization never recovered and slipped into oblivion at this time.

It would be an overstatement to argue that influenza alone destroyed the mission of the Floating Christian Endeavor. The massive numbers and logics related to World War I overwhelmed Jones’ organization. The rise of the Young Men’s Christian Association and its work, along with the growth of the naval chaplaincy, was clear competition, as seen from reading through Christian Endeavor material of the time. However, the impact of influenza was probably much greater than just affecting the central leadership and the death of Jones herself. It is unknown how many Floating Christian Endeavor societies onboard ships and in ports across the East Coast were impacted by the epidemic, but since it spread primarily through sailors from the beginning, its role in the demise of the organization as a whole was most likely significant. The Reverend AC Crews noted at the time:

There can be no doubt about it; the prevailing influenza has interfered very seriously with church work of all kinds, as indicated by the withdrawal of public services in many places and the stoppage of Sunday-school and Christian Endeavor activities. (Crews, 1919)

In a report to the Boston Seaman’s Friend Society, Jones’ co-founder, Madison Edwards, noted of his friend:

The large ships of war in the English, German, and United States navies have their little bands of Floating Christian Endeavorers. Miss Jones corresponded with all of these, and had vital interest in every one of them. The work in Japan was as familiar to her as the work in the homeland. She was most devoted to her work, and that without compensation. (Edwards, 1919)

World War I ended in November 1918 but, as people began to celebrate Armistice Day, the virus continued to spread. Mortality rates were high among the 20–40 age group, as well as the young and old, and it is estimated that 675,000 people died in the USA from the influenza epidemic. While individual ministries to sailors, such as Madison Edwards’ Seaman’s Bethel on Martha’s Vineyard, continued to work with sailors (see Wiseman, *c.*^1978^), and a form of the Floating Christian Endeavor even continued to operate in the British navy for a time, the central organization Jones provided was lost and never recovered. No one appears to have been trained to replace her and, given the general confusion at the end of World War I and the influenza epidemic, no one stepped forward to fill her role. The pandemic had sadly claimed another victim—the vibrant mission of the Floating Christian Endeavor.

## Influenza and the ministry of John R Mott in 1918

### Benjamin L Hartley

For readers of this journal, the name John R Mott is most commonly associated with the work of the Student Volunteer Movement, Young Men’s Christian Association (YMCA), World Student Christian Federation, and International Missionary Council at the end of the 19th century and beginning of the 20th century. As Robert Danielson noted above, the YMCA was involved in a massive effort during World War I to minister among US soldiers and in prisoner-of-war camps throughout Europe. Seven thousand YMCA volunteers served during the war in France, Romania, Turkey, Russia, north and east Africa, and elsewhere (Copeland and Xu, 2018: 5). When the deadliest second wave of the Spanish flu began to spread in the spring of 1918, the World War was showing signs that an end to its scourge was in sight, but the victims of influenza were just beginning to grow in number.

Influenza most directly impacted John R Mott when, on 24 September 1918, his nephew, Private Ralph M McAdam of the Marine Corps, was one of the 23 soldiers who died of the flu onboard a ship sailing to France. The war ended before any of the other men on the ship arrived on the front lines of the conflict. Mott corresponded with the chaplain about his nephew’s final hours and arranged for a private funeral in Hartford, Connecticut. Mott counseled his sister, Alice, not to travel to Hartford for her son’s funeral because of the pandemic.

A month later, Mott received news that Joseph Oldham, one of his best friends and his co-laborer on the Edinburgh 1910 Continuation, also had a severe case of influenza. By the middle of November, however, Oldham was once again hard at work trying to figure out how German mission territories in Africa might be best served after the war. Oldham (1918) wrote Charles R Watson (who, a few months later, accompanied Mott in representing North American mission boards’ interests at the Paris Peace Conference) a letter filled with questions about how mission organizations and churches should minister in these new areas, while acknowledging that “we cannot hand about native Christians as if they were property, or transfer them to a different ecclesiastical allegiance without consulting their wishes.” Oldham mentioned that a “round table conference” was needed to sort out fully the many competing interests and missiological priorities, but that until that could happen, some temporary decisions needed to be made. It is likely that the influenza pandemic was at least one of the factors preventing such a meeting from taking place.

Mott and Watson finally sailed for the Paris Peace Conference on 16 March 1919, but shortly after arriving in Paris Mott got sick with a mild case of the flu and was hospitalized for a few days. He held meetings from his hospital room and reported in a letter home to his wife, Leila, that while in the hospital he “spent very busy and profitable days” (Hopkins, 1979: 562). One of the people who met with Mott in his hospital room was World Student Christian Federation and YMCA leader Wang Zhengting (also called Chengting Wang), who was the head of the Chinese delegation at the Peace Conference. In Mott’s hospital room, Wang probably discussed the importance of China gaining control of the Shandong province, which had been under German control during the war. He wrote to Mott a week later when they were both in France, underscoring how critical this was (Wang, 1919a).

Mott was unable to meet with President Wilson when they were both in France but was able to speak with a member of Wilson’s team in Paris, Colonel House. For a variety of reasons, however, Mott was unable to exert much influence over his friend President Wilson, and Chinese hopes (as well as the hopes of many others inspired by Wilson’s rhetoric of “self-determination”) of regaining control of Shandong were met with disappointment by the end of April. One can only speculate as to whether the influenza pandemic was a factor in Mott’s failure to convince President Wilson to give Shandong province back to China. Wilson’s biographer notes that his decision not to do so was “the most anguished choice” of the months-long Paris Peace Conference (Cooper, 2009: 493). Chinese cities erupted in protest as a result, in what has become known as the May Fourth Movement. Wang (1919b) wrote Mott a heartbreaking letter, stating: “April 30^th^ will go down in the history of China as a dark day . . . All cry of race equality was, as I told you, a smoke-screen.”

By contrast, John R Mott’s greatest achievement in the face of the influenza pandemic of 1918–1919 was his ability to persist in a massive fundraising campaign to support US soldiers. Shortly after the USA entered the war in April 1917, Mott became chairperson of the seven-organization United War Work Council, which was intended to coordinate service to US soldiers (Matthews, 1932).^1^ The Council was ready to launch a massive US$200 million fundraising campaign when the influenza pandemic began to infect millions.

President Wilson, along with Wall Street financiers who were on the fundraising committee, including John D Rockefeller Jr, urged Mott to postpone the campaign. After a brief meeting with Mott in New York, businessman George W Perkins left Mott’s office in a huff: “The whole outfit can go to hell,” he fumed. “The egotism of that man beats everything. He thinks he knows more than the President of the United States and all the bankers in Wall Street. I am through.” A few minutes later, Jewish businessman Mortimer Schiff also emerged from Mott’s office, but with a smile. He reported what Mott asked of him: “Your people for generations back, and I know them well, have always been fundamentally religious. Will you not prayerfully meditate on this problem and on getting John D. Rockefeller and Mr. Perkins to open their minds again?” By the end of the day, the bankers had come round to Mott’s side. The campaign was launched and exceeded their goal with US$225 million raised (Matthews, 1932). The funds were used to provide pastoral care and physical assistance of various kinds to soldiers as they gradually returned home.

These stories from the life of John R Mott illustrate that the pandemic of 1918 affected Mott and the friends and movements he influenced in a variety of ways. Mott grieved the loss of a dear nephew and was perhaps inhibited in the work he was doing with Joseph Oldham to plan for a post-war world. One may speculate that Mott’s failure to sway Wilson to agree to the Chinese delegation’s lobbying efforts may have also been partly due to Mott’s illness. Mott’s persistence in continuing with plans for the United War Work Council’s US$200 million campaign is an example of acknowledging the challenge of his context but nonetheless working to overcome it through the art of persuasion and prayer.

## Conclusion

The three essays above provide several important lessons for missiologists to consider in continuing to grapple with the direct and indirect effects of the COVID-19 pandemic. Perhaps the most important lesson to derive from these examples of mission during the 1918–1919 influenza pandemic is that, in these times of uncertainty, it is especially important to spiritually discern how missiologists should respond to the many different crises around them. In some of the contexts where we serve, this may very well be a time for initiating new ministries (like Aladura) or persisting with fundraising plans, even if the naysayers advise a posture of retrenchment (like Mott). In other cases, of course, our current pandemic may indeed lead to the end of mission initiatives which many have held close to their heart for a long time. God’s mission, however, continues, and we are privileged to be prayerful participants in that, regardless of our circumstances.

## Notes

1. The United War Work Council included the participation of the Jewish Welfare Board, the Knights of Columbus, the Salvation Army, the YMCA and Young Women’s Christian Association, the American Library Association, and the Playground Association.
